# Characteristics of indications, clinical trial evidence, clinical benefits and the costs of price-negotiated multi-indication drugs for solid tumours in China

**DOI:** 10.7189/jogh.15.04121

**Published:** 2025-05-09

**Authors:** Pei Zhang, Mengdie Zhang, Rui Ma, Jingxuan Wei, Yuwen Bao, Lingli Zhang, Xiaodan Qian, Dan Su, Xin Li

**Affiliations:** 1 Department of Pharmaceutical Regulatory Science and Pharmacoeconomics, School of Pharmacy, Nanjing Medical University, Nanjing, China; 2 Department of Health Policy, School of Health Policy and Management, Nanjing Medical University, Nanjing, China; 3 School of International Pharmaceutical Business, China Pharmaceutical University, Nanjing, China; 4 Department of Pharmacy, The Second People's Hospital of Changzhou, the Third Affiliated Hospital of Nanjing Medical University, Changzhou, China

## Abstract

**Background:**

Cancer is a leading cause of death globally, with China accounting for a significant portion of new cases and deaths. The Government of China has introduced the National Drug Price Negotiation (NDPN) policy to mitigate the high costs of anticancer drugs, especially multi-indication drugs, aimed at improving patient access to effective treatments.

**Methods:**

In this retrospective study, we examined 24 multi-indication anticancer drugs for solid tumours included in the National Reimbursement Drug List (NRDL) between 2016 and 2023. We collected characteristics of indication, clinical trial evidence, and clinical benefits of these drugs, calculated monthly drug costs, and assessed the associations the two by regression and correlation analyses.

**Results:**

We observed a significant reduction in the median monthly drug cost from USD 3863.08 before NDPN to USD 732.91 after their inclusion in the NRDL. However, the correlation analyses showed no significant relationships between drug costs and characteristics of indications, clinical trial evidence, and clinical benefits, while American Society of Clinical Oncology Value Framework scores demonstrated a negative correlation with costs, indicating that pricing may not accurately reflect clinical benefits.

**Conclusions:**

While we found that the NDPN policy has significantly reduced drug costs, we did not observe a significant correlation between costs and specific characteristics. This highlights a need for a more transparent pricing mechanism linked to clinical efficacy to improve the accessibility and affordability of cancer therapies that effectively balance the interests of pharmaceutical companies, patients, and health insurance funds.

Cancer is the second leading cause of death globally in terms of disability-adjusted life years and years of life lost [1]. According to the Global Cancer Observatory (GLOBOCAN) 2020, China accounted for 24% of all new cancer cases and 30% of cancer-related deaths globally [2]. Cancer mortality and DALY rates in the country are significantly higher than those in the USA and the UK due to its large population, uneven regional development, and relatively backwards cancer control strategies. This burden, which is expected to rise in the coming decade, is largely driven by China’s ageing population and unhealthy lifestyles [3].

To address the strong demand for anticancer drugs among Chinese patients, the Government of China has implemented a range of policy measures aimed at streamlining all stages from drug development to market sales. Among them, the National Drug Price Negotiation (NDPN) policy [4–6] is an important measure aimed at addressing the lack of market competition and high pricing of innovative drugs or patented drugs with definite efficacy. By 2023, eight rounds of the NDPN had been conducted, with anticancer drugs consistently representing 20% or more of the medicines subject to price negotiation [7]. Some of these drugs are considered categorised as ‘multi-indication’ drugs, as they are effective for multiple conditions or disease stages (first, second, and third lines of treatment). Establishing pricing or reimbursement amounts for them is challenging due to the variations in clinical efficacy and patient populations across different indications.

Previous studies have explored the correlation between costs and factors such as efficacy, safety, and the delay between marketing and health insurance enrolment for multi-indication anticancer drugs in Australia, the USA, and certain European countries [8–11], However, there has been no comprehensive evaluation of the correlation between the costs of price-negotiated multi-indication cancer drugs and these factors in China. We thus aimed to compare the relationship between monthly drug costs and indication characteristics, clinical trial evidence, and clinical benefits of price-negotiated multi-indication cancer drugs for solid tumours, both before and after their inclusion in the National Reimbursement Drug List (NRDL) through the NDPN.

## METHODS

### Study design and objectives

We designed this retrospective analysis to evaluate the economics of multi-indication anticancer drugs for solid tumours included in the NRDL through the NDPN between 2016 and 2023. The primary objective was to compare the correlation between monthly drug costs and factors such as indication characteristics, clinical trial evidence, and clinical benefits of price-negotiated multi-indication cancer drugs for solid tumours. One researcher (ZP) was responsible for collecting and organising the drug information involved in this study, and then two others (ZP and ZMD) simultaneously collected and collated the raw data against the drug information using Microsoft Excel, version 2021 (Microsoft Corporation, Redmond, WA, USA) to improve the efficiency and accuracy of the analysis. If any outliers are identified during data processing, they were reviewed and resolved by a senior reviewer (LX), ensuring consistency and reliability across the data.

### Data sources

We retrieved information from the NRDL, the Center for Drug Evaluation (CDE) of the National Medical Products Administration, and clinical trial registries (ClinicalTrials.gov and chinadrugtrials.org). We further used secondary data for our analyses, including published clinical trial literature and clinical trial data from drug inserts.

### Inclusion and exclusion criteria

We selected anti-cancer multi-indication drugs for solid tumours that were included in the NRDL through NDPN between 2016 and 2023. We excluded haematological tumour drugs because their therapeutic effects and mechanisms of action differ significantly from those of solid tumours, and because including such drugs may interfere with the research results. We also excluded traditional Chinese medicine due to its complex composition and lack of standardisation, which could have led to inconsistencies and lack of comparability in efficacy assessment. Finally, to ensure a focus on primary therapeutic drugs only, we excluded drugs used for auxiliary treatments and maintenance therapies.

For each indication, we search for relevant clinical trials of target anti-cancer drugs on the ClinicalTrials.gov website using the drug name and indication as search criteria. We obtain the corresponding clinical trial numbers and retrieved published clinical trial literature for those indications. For indications lacking registered clinical trials on ClinicalTrials.gov, we supplemented our search with the drug labelling. Since one indication might have corresponded to multiple clinical trials, we implemented a secondary screening for the acquired clinical trials to ensure the scientific validity and applicability of the research data. Specifically, we included phase III and high-quality phase II clinical trials, focussing on randomised controlled trials (RCTs) and excluding single-arm, adjuvant therapy, and maintenance therapy trials. We included trials based on Chinese or East Asian populations, including only those international multicentre clinical trials which had 20% or more of the Chinese or East Asian populations, with a balanced distribution between the treatment and control groups. All clinical trials in our analysis had to have progression-free survival (PFS) and/or overall survival (OS) as the primary endpoints, with complete clinical efficacy results for at least one of these endpoints. If there were multiple qualifying clinical trials for the same drug, we included those that were most aligned with the indication and focus on the Chinese or Asian populations or that had the best clinical outcomes. A total of 24 anti-cancer drugs corresponding to 58 indications were finally included (Figure S1 in the **Online Supplementary Document**). Data collection was completed on 31 July 2024.

### Characteristics of multi-indication anticancer drugs

We collected data on each drug, including the number of indications, innovation status, mechanism of action, and molecule type (Table S1 in the **Online Supplementary Document**). Specifically, we determined the number of indications based on that registered in the NRDL, assessed innovation status based on the classification provided on the website of the CDE under the National Medical Products Administration [12] categorised the mechanisms of drug action into three categories (cytotoxic chemotherapy, targeted therapy, and immune checkpoint inhibitors [13]), and grouped molecule types as small molecules, monoclonal antibodies, immune checkpoint inhibitors, and antibody-drug conjugates [14].

### Characteristics of indications

For each indication, we recorded the marketing approval date by the CDE, the inclusion date in the NRDL (January of the year the indication became formally available), the type of therapy (monotherapy *vs*. combination therapy), line of therapy (first-line, second-line, or third-line), and type of marketing approval (regular approval, priority review and approval, special approval, or conditional approval) (Table S2 in the **Online Supplementary Document**).

### Clinical trial evidence

For clinical trial evidence, we collected data on the number of patients enrolled, staging (stage II/III vs stage III), blinding (open-label vs double-blind), and the outcomes of clinical trials for each indication (Table S3 in the **Online Supplementary Document**).

### Clinical benefits

We extracted the median OS and median PFS of the experimental and controlled groups from clinical trials. We used the percentage difference in median OS and PFS between the two treatment regimens as the traditional measure of clinical benefits, expressed as the incremental percentage of OS (∆OS%) and incremental percentage of PFS (∆PFS%), respectively (Table S4 in the **Online Supplementary Document**). For some clinical trials where the OS data were immature and lacked ∆OS% data, we employed the mean imputation method to handle the missing values, ensuring the completeness of the analysis and the reliability of the results. The formulas for calculating ∆OS% and ∆PFS% are as follows:

ΔOS% = (*median OS* (*months*) *in experimental group* − *median OS* (*months*) *in control group*)/(*median OS* (*months*) *in control group*)

ΔPFS% = (*median PFS* (*months*) *in experimental group* − *median PFS* (*months*) *in control group*)/(*median PFS* (*months*) *in control group*)

We used two established value frameworks from the American Society of Clinical Oncology (ASCO) and the European Society for Medical Oncology (ESMO) to convert the extracted OS and PFS data into intuitive efficacy scores (Table S4 in the **Online Supplementary Document**). The ASCO Value Framework (ASCO-VF) version 2.0 employs a 100-point scale that assesses treatment benefits, toxicity, bonus points, and net health benefit based on cancer progression [15,16]. The ESMO Magnitude of Clinical Benefit Scale (ESMO-MCBS) version 1.1 scores range from 1 to 5 [17,18] and evaluates the type of study, clinical endpoints, and benefits of the control drug. ASCO-VF scores in our study were calculated based on clinical trial results. ESMO-MCBS scores were directly adopted from ESMO scorecards when available. If not available, we calculated these values on our own.

### Drug prices

We obtained the prices of multi-indication cancer drugs for solid tumours before and after NDPN from the winning bid prices provided by the Government of China, which were the prices at which hospitals, medical centres, and other medical institutions purchased these drugs. However, some multi-indication cancer drugs for solid tumours have separate medical insurance payment standards, drug price standards, quantity standards and reimbursement ratio standards after NDPN. Regardless of how the updated prices change, we only measured the initial price of each drug registered in the NRDL.

We calculated monthly drug costs for the corresponding indications of multi-indication cancer drugs for solid tumours based on a 28-day treatment cycle [19] by combining the drug dosage for each indication listed in the drug insert and the medication regimen from clinical trials. We calculated the dosage for an adult with an average body surface area of 1.72 m^2^ and an average weight of 65 kg [20]. If the initial dose differs from the subsequent doses, the average dose per cycle is used as the standard. We finally adjusted all monthly drug costs for medication regimens to a four-week price. When multiple approved dosages are available (*e.g.* 50mg per tablet and 100mg per tablet), we selected the dosage with the lowest cost per unit.

### Statistical analyses

We used descriptive statistics to characterise our baseline sample. We conducted linear univariate ordinary least squares regression analyses and multiple linear regression analysis to evaluate relationships between collected variables and monthly treatment costs, with monthly treatment costs treated as the dependent variable, assigning values to each variable for ease of analysis (Table S5 in the **Online Supplementary Document**). We analysed non-normally distributed continuous variables using Spearman’s rank correlation coefficient. We visualised our results with scatter plots generated in Microsoft Excel, version 2021 (Microsoft Corporation, Redmond, WA, USA) and performed all statistical analyses in SPSS, version 23.0 (IBM Corporation, Armonk, NY, USA). *P*-values <0.05 indicated statistical significance.

## RESULTS

### Sample overview

Our study included 24 multi-indication cancer drugs for solid tumours, covering 58 indications (Figure S1 in the **Online Supplementary Document**). Of these, 10 were innovator (41.67%) and 14 were non-innovator drugs (58.33%). Twenty drugs (83.33%) were targeted therapies, while four (16.67%) were immunosuppressive, with 17 (70.83%) classified as small molecules and two (8.33%) as monoclonal antibodies. The median monthly drug cost was USD 3863.08 (interquartile range (IQR) = 2890.64–5617.42) before inclusion into the NRDL and dropped to USD 732.91 (IQR = 491.84–1456.03) after inclusion, reflecting a significant reduction. The Spearman correlation (**Table 1**) and univariate regression analyses (**Table 2**) showed that some factors have statistically significant relationships with costs, while the results of multiple linear regression analysis were not statistically significant (**Table 3**).

**Table 1:** Results of Spearman's correlation analysis by influencing factors

	Spearman's correlation coefficient (before entering NRDL)		Spearman's correlation coefficient (after entering NRDL)	
	**r_s_**	***P*-value**	**r_s_**	***P*-value**
**Characteristics of indications**				
Delay between marketing and enrolling in NRDL	0.150	0.260	0.371	0.004
Lines of therapy	−0.062	0.644	0.272	0.039
Type of approval for indications	0.019	0.885	0.322	0.014
Type of treatment	−0.052	0.700	−0.401	0.002
**Characteristics of clinical trials**				
Number of enrolled patients	0.269	0.041	0.068	0.612
Staging of clinical trials	0.386	0.003	0.218	0.100
Blindness	0.127	0.344	0.115	0.391
**Clinical benefits**				
∆OS%	−0.139	0.404	−0.187	0.261
∆PFS%	−0.009	0.949	0.185	0.165
ASCO-VF score	−0.283	0.031	−0.169	0.204
ESMO-MCBS score	−0.132	0.323	−0.179	0.178

NRDL – National Reimbursement Drug List, ΔOS% – percentage improvement of overall survival, ΔPFS% – percentage improvement of progression-free survival, ASCO-VF – American Society of Clinical Oncology – Value Framework, ESMO-MCBS – European Society for Medical Oncology – Magnitude of Clinical Benefit Scale

**Table 2:** Results of one-way regression analyses

			Before enrolling in NRDL			After enrolling in NRDL		
	**β (95% CI)**	***P*-value**		**R^2^**	**β (95% CI)**		***P*-value**	**R^2^**
**Characteristics of indications**								
Delay between marketing and enrolling in NRDL	0.294 (0.0382, 0.550)	0.025		0.070	0.123 (0.142, 0.633)		0.003	0.136
Lines of therapy	−0.078 (−0.345, 0.188)	0.558		−0.012	0.139 (−0.126, 0.403)		0.298	0.002
Type of approval for indications	−0.036 (−0.303, 0.232)	0.790		−0.017	0.018 (−0.248, 0.285)		0.891	−0.018
Type of treatment	−0.027 (−0.295, 0.240)	0.839		−0.017	−0.254 (−0.511, 0.004)		0.054	0.048
**Characteristics of clinical trials**								
Number of enrolled patients	0.343 (0.092, 0.595)	0.008		0.102	0.387 (0.139, 0.631)		0.003	0.134
Staging of clinical trials	0.235 (−0.025, 0.495)	0.075		0.039	0.175 (−0.089, 0.437)		0.190	0.013
Blindness	0.220 (−0.041, 0.481)	0.097		0.031	0.058 (−0.208, 0.324)		0.664	−0.014
**Clinical benefits**								
∆OS%	−0.078 (−0.479, 0.299)	0.642		−0.022	−0.145 (−0.524, 0.207)		0.384	−0.006
∆PFS%	−0.122 (−0.388, 0.361)	0.144		−0.003	−0.051 (−0.317, 0.215)		0.703	−0.015
ASCO-VF score	−0.267 (−0.525, 0.009)	0.043		0.055	−0.168 (−0.430, 0.095)		0.207	0.011
ESMO-MCBS score	−0.085 (−0.351, 0.182)	0.527		−0.011	−0.191 (−0.452, 0.072)		0.152	0.019

CI – confidence interval, NRDL – National Reimbursement Drug List, ΔOS% – percentage improvement of overall survival, ΔPFS% – percentage improvement of progression-free survival, ASCO-VF – American Society of Clinical Oncology – Value Framework, ESMO-MCBS – European Society for Medical Oncology – Magnitude of Clinical Benefit Scale

**Table 3:** Results of multiple regression analysis

	Unstandardised coefficient		Standardised coefficient			Covariance statistics	
	***β***	**SD**	***β***	***T***	***P*-value**	**Tolerance**	***VIF***
**Before enrolling in NRDL**							
Constant	−8298.682	6164.023	-	−1.346	0.190		
Characteristics of indications							
*X_1_: Delay between marketing and enrolling in NRDL*	130.274	108.759	0.200	1.198	0.242	0.806	1.241
*X_2_: Lines of therapy*	−701.898	891.594	−0.172	−0.787	0.438	0.467	2.140
*X_3_: Type of approval for indications*	−637.891	714.939	−0.205	−0.892	0.380	0.422	2.367
*X_4_: Type of treatment*	127.060	1261.519	0.021	0.101	0.921	0.499	2.005
Characteristics of clinical trials							
*X_5_: Number of enrolled patients*	5.027	2.541	0.347	1.978	0.059	0.726	1.377
*X_6_: Staging of clinical trials*	2696.672	1325.159	0.355	2.035	0.052	0.735	1.360
*X_7_: Blindness*	2726.104	1321.166	0.466	2.063	0.049	0.439	2.276
Clinical benefits							
*X_8_: ∆OS%*	5912.580	3809.923	0.315	1.552	0.133	0.543	1.843
*X_9_: ∆PFS%*	16.903	8.562	0.514	1.974	0.059	0.330	3.029
*X_10_: ASCO-VF score*	−83.276	49.986	−0.358	−1.666	0.108	0.486	2.057
*X_11_: ESMO-MCBS score*	33.039	657.623	0.011	0.050	0.960	0.475	2.106
**After enrolling in NRDL**							
Constant	−570.498	2332.973	-	−0.245	0.809		
Characteristics of indications							
*X_1_: Delay between marketing and enrolling in NRDL*	111.814	41.163	0.469	2.716	0.012	0.806	1.241
*X_2_: Lines of therapy*	−118.283	337.453	−0.079	−0.351	0.729	0.467	2.140
*X_3_: Type of approval for indications*	88.157	270.592	0.078	0.326	0.747	0.422	2.367
*X_4_: Type of treatment*	−419.888	477.463	−0.193	−0.879	0.387	0.499	2.005
Characteristics of clinical trials							
*X_5_: Number of enrolled patients*	2.343	0.962	0.443	2.436	0.022	0.726	1.377
*X_6_: Staging of clinical trials*	231.673	501.549	0.083	0.462	0.648	0.735	1.360
*X_7_: Blindness*	−42.190	500.038	−0.020	−0.084	0.933	0.439	2.276
Clinical benefits							
*X_8_: ∆OS%*	855.248	1441.989	0.125	0.593	0.558	0.543	1.843
*X_9_: ∆PFS%*	1.755	3.240	0.146	0.542	0.593	0.330	3.029
*X_10_: ASCO-VF score*	−8.223	18.919	−0.097	−0.435	0.667	0.486	2.057
*X_11_: ESMO-MCBS score*	29.612	248.899	0.027	0.119	0.906	0.475	2.106

SD – standard deviation, VIF – Variance Inflation Factor, NRDL – National Reimbursement Drug List, ΔOS% – percentage improvement of overall survival, ΔPFS% – percentage improvement of progression-free survival, ASCO-VF – American Society of Clinical Oncology – Value Framework, ESMO-MCBS – European Society for Medical Oncology – Magnitude of Clinical Benefit Scale

### Indication characteristics

For 58 indications, the median delay between marketing and enrolling in NRDL was 1.80 years (IQR = 1.09–3.08). Of these, 32 (55.17%) were regular approvals, 13 (22.41%) were priority reviews and approvals, nine (15.52%) were special approvals, and four (6.90%) were conditional approvals. Of the approved therapies, 29 (50.00%) were combination therapies and 29 (50.00%) were monotherapies. There were 58 indications, of which 37 (63.79%) were for first-line therapy, 16 (27.59%) were for second-line therapy, and five (8.62%) were for third-line therapy.

The correlation analysis showed that after inclusion into the NRDL, the delay between marketing and enrolment (r_s_ = 0.371, *P* = 0.004), therapy lines (r_s_ = 0.272, *P* = 0.039), and approval type (r_s_ = 0.322, *P* = 0.014) were positively correlated with monthly costs, while therapy types showed a negative correlation (r_s_ = −0.401, *P* = 0.002). Univariate regression results indicated a positive correlation between the delay and costs, with coefficients of 0.29 (95% confidence interval (CI) = 0.142–0.633, *P* = 0.025, R^2^ = 0.07) before inclusion into the NRDL and 0.12 (95% CI = 0.142–0.633, *P* = 0.003, R^2^ = 0.14) after inclusion.

### Clinical trials evidence

All clinical trials were RCTs, with 51 (87.93%) being phase III, including 36 double-blind trials (62.07%) and 22 open-label trials (37.93%). The median number of patients enrolled in these trials was 449 (IQR = 360–614).

The correlation analysis suggested that before inclusion into the NRDL, clinical trial phases (r_s_ = 0.269, *P* = 0.041) and the number of patients enrolled (r_s_ = 0.386, *P* = 0.003) were positively correlated with monthly costs, while other factors showed no correlation (**Table 1**). Univariate regression results indicated a positive correlation between the number of enrolled patients and costs, with coefficients of 0.343 (95% CI = 0.092–0.595, *P* = 0.008, R^2^ = 0.102) before inclusion into the NRDL and 0.387 (95% CI = 0.139–0.631, *P* = 0.003, R^2^ = 0.134) after inclusion (**Table 2**).

### Clinical benefits

All 58 indications had mature PFS results, with 38 also reporting mature OS results. The ΔOS% ranged from −4.98% to 62.26%, while the ΔPFS% varied from −23.81% to 485.71%. The median ΔOS% was 27.72% (IQR = 21.46–36.5), and the median ΔPFS% was 51.35% (IQR = 24.38–85.98). ASCO-VF scores ranged from −12.80 to 79.20, with a median score of 37.95 (IQR = 28.63–50.82). ESMO-MCBS scores ranged from 1 to 4, and the median score was 4 (IQR = 3–4).

**Figure 1:**
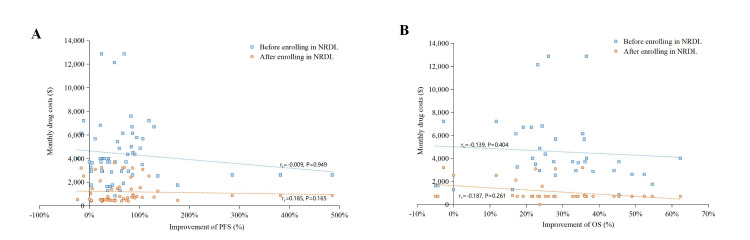
Correlation between monthly costs and percentage improvement among indications. **Panel A.** Value assessment of ΔPFS%. **Panel B.** Value assessment of ΔOS%. ΔPFS% – percentage improvement of progression-free survival, ΔOS% – percentage improvement of overall survival, NRDL – National Reimbursement Drug List.

**Figure 2:**
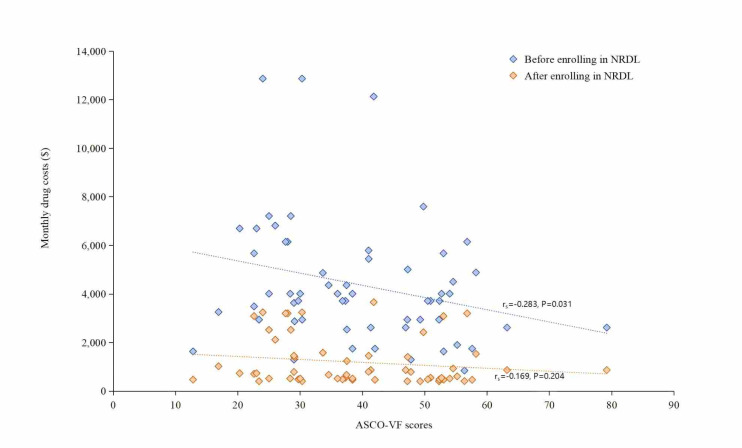
Correlation between monthly costs and ASCO-VF scores among indications. ASCO-VF score – American Society of Clinical Oncology-Value Framework, NRDL – National Reimbursement Drug List.

The correlation analysis showed that before inclusion into the NRDL, neither ΔPFS% (r_s_ = −0.009, *P* = 0.949) nor ΔOS% (r_s_ = −0.139, *P* = 0.404) correlated with monthly costs, which remained true after inclusion (ΔPFS%: r_s_ = 0.185, *P* = 0.165 and ΔOS%: r_s_ = −0.187, *P* = 0.261) (**Figure 1**). Among the two scale frameworks, only the ASCO-VF score had a significant negative correlation with monthly costs (r_s_ = −0.283, *P* = 0.031) before inclusion into the NRDL (**Figure 2**). Univariate regression results also indicated a negative correlation between the ASCO-VF scores and costs before NRDL enrolment, with a coefficient of −0.267 (95% CI = −0.525, 0.009, *P* = 0.043, R^2^ = 0.055) (**Table 2**).

## DISCUSSION

To our knowledge, this is the first study of the relationship between costs, indication characteristics, clinical trial evidence, and clinical benefits of multi-indication cancer drugs for solid tumours in the context of the NDPN's implementation in China. We analysed 24 multi-indication cancer drugs for solid tumours that were included in the NRDL through the NDPN between 2016 and 2023, encompassing 58 indications. Overall, our findings indicate a reduction in drug prices following the NDPN. However, correlation and univariate regression analyses did not reveal any significant relationships between costs and specific characteristics; in fact, we observed a negative correlation between drug costs and indicators related to clinical effectiveness.

For indication characteristics, after being included in the NRDL, we observed a positive correlation between the delay from marketing to inclusion into the NRDL, lines of therapy, market approval type, and costs, while costs were negatively correlated with the type of therapy. The longer the delay from marketing to enrolment in the NRDL, the more time the drug's efficacy in treating a particular indication is validated by the market, and the more real-world data can be referenced to support its effectiveness; First-, second-, and third-line treatments are different treatment regimens based on treatment stage and severity of the disease [21,22]. First-line treatment is the most effective and has the fewest side effects, making it the initial treatment option after diagnosis. It is also more cost-effective and thus is more likely to be included in the NRDL, allowing patients to access it at lower prices. In our study, the proportion of indications for first-line treatment exceeded 60%. Second-line treatment is the treatment regimen used after the failure of first-line treatment. It may have inferior efficacy, more side effects, or be more expensive than first-line treatment. Third-line treatment refers to the treatment options used after second-line treatment has failed. Therefore, as the number of treatment lines increases, the available effective treatment options decrease, leading to higher requires more research and development costs as well as increased drug costs; According to the assignment (Table S5 in the **Online Supplementary Document**), the costs of special approval indications was higher, particularly for conditional approval marketing indications. Conditional approval is a registration process in which the National Medical Products Administration encourages innovation in clinical benefit-oriented medicines and accelerates the marketing of urgently needed medicines with outstanding clinical benefit [22,23], which are typically of more prominent clinical benefit and correspondingly more expensive. Monotherapy costs are higher due to high development and production costs and exclusive market position [24,25]. In contrast, combination therapies are less expensive, possibly because they use several existing drugs with relatively low production costs, and because combination therapies can improve therapeutic efficacy and reduce cycle time or dosage requirements, while the highly-competitive market for combination therapies helps keep prices down.

For clinical trial evidence, before inclusion into the NRDL, the phase of clinical trials and the number of patients enrolled were positively correlated with costs; after inclusion, the number of patients enrolled was positively correlated with costs. Phase III clinical trials are the confirmatory phase, designed to further validate the therapeutic effect and safety of the medicine in patients with the target indications, assess the risk-benefit balance, and ultimately provide sufficient evidence to support regulatory submissions. The sample size is much larger than in the previous two phases of the trial. A larger sample size helps to provide more comprehensive data on the safety and efficacy of the drug, making the results of this phase more credible [26,27]; The larger the number of patients enrolled, the less bias in the results due to random error, and the higher the statistical significance and confidence of the trial results [27,28], resulting in higher quality clinical trial results and higher drug prices. While the studies indicate that a larger sample size is more helpful in providing comprehensive data on drug safety and efficacy, simply increasing the sample size cannot resolve the issues at hand. Instead, more refined research protocols should be designed that focus on the characteristics of specific types of cancers or patient populations in order to obtain more accurate and representative clinical trial data, which can serve as a basis for drug pricing.

For clinical benefits, only the ASCO-VF score negatively correlated with the monthly costs before and after inclusion into the NRDL. This suggests that the pricing of price-negotiated drugs is not based on the clinical benefits of each indication, and that some high-efficacy indications are associated with lower costs. This reflects a deficiency in the application of pharmacoeconomic evaluations in the NDPN process. The weak association between the costs of multi-indication anti-cancer drugs and the expected treatment expenses before and after national negotiations may have two main reasons. First, the negotiation process is a contest between the government (driven by patient interests) and enterprises (which are profit-driven). The pricing is often influenced by market supply and demand, research and development costs, and competitive environments, rather than being solely based on the clinical efficacy of the drugs. This means that even if certain drugs demonstrate high clinical efficacy for a specific indication, their pricing may diverge from this clinical efficacy, driven by commercial interests. Second, for multi-indication drugs, the market entry times for different indications may vary. They can be categorised into initial indications and new indications. Currently, China employs a fixed-price pricing method, where the degree of price reduction after negotiation is partially dependent on the initial indication when the drug enters the reimbursement list. This leads to a weak correlation between new indications and drug costs. Our results are not an isolated case, as studies in several countries have found no correlation between drug efficacy and treatment costs. For example, two studies on PD-1/PD-L1 inhibitors and new anti-solid tumour drugs to be included in NRDL from 2016 to 2020 in China concluded that drug prices were independent of the level of clinical benefit, based on the ASCO-VF and ESMO-MCBS scores [29,30]. Bao and colleagues [31] examined the correlation between the cost and clinical benefits of price-negotiated new drugs for lung and breast cancers, and observed that the negotiation policy reduced the cost of the drugs, but did not generate the expected correlation between the value and costs. Vokinger and colleagues [32] compared the USA and four European countries (UK, Switzerland, Germany, and France) and found that a positive correlation between ASCO-VF scores and the cost of cycle therapy in France only. Zhang and colleagues [33] analysed the relationship between clinical benefits and prices of anticancer drugs in four rounds of national price negotiations from 2017 to 2020, and noted no significant correlation between the daily drug cost of anticancer drugs and their clinical benefits after implementation of the negotiation policy. In Japan, clinical benefit is also mainly based on PFS and OS, and relevant studies have shown a lack of correlation between the costs of complete treatment cycles of anticancer drugs and clinical benefits in Japan [34,35].

In sum, although we found no significant relationship between the cost of treatment and the characteristics of multi-indication drugs, our results can provide a valuable reference for the existing drug pricing models, particularly for multi-indication anti-cancer drug pricing models, which will assist health insurance authorities in formulating more reasonable pricing strategies. Furthermore, our findings indicate that drug prices are not being linked to their efficacy. It is recommended that the research methods for the application of pharmacoeconomics be strengthened in the national drug price negotiation system. The price of multi-indication anti-cancer medicines should be determined by referring to the practices of developed Western countries, using the primary indication (the one with the largest number of patients) as the basis for setting the price. This approach would change the current practice of lowering the price of cancer medicines simply by adding a new indication in the NRDL. The goal is to link the price of drugs to their actual efficacy, so that even if the price increases, as long as the incremental cost-effectiveness ratio is below the threshold compared to other medicines, it will be considered economically viable. Ultimately, the efficacy indicators of the multi-indication anti-cancer medicine should play a key role in determining its price. Furthermore, in order to optimise the pharmaceutical pricing system, the Government of China can reassess whether the current drug pricing affects patient accessibility and affordability, thus allowing for policy adjustments that promote the establishment of a fairer health care system.

### Limitations

This study has some limitations. First, we included only solid tumour indications, excluding haematological drugs and traditional Chinese medicine, as well as single-arm clinical trials from the analysis. This means that our results may be biased and the conclusions regarding the correlation between treatment costs, clinical efficacy and indication characteristics, may be overstated. Second, OS is considered a valid clinical endpoint for drug approval, but due to the lack of maturity of OS data from clinical trials for some of the indications for which it is used, the value framework is based on PFS as a surrogate benefit when conducting clinical benefit assessments. Currently, the relationship between the early endpoint of PFS and OS is not fully clarified, which may have affected the accuracy of our results. Third, we obtained the clinical trial information from drug inserts downloaded from the CDE website or data from the ClinicalTrials.gov website; this means they were not uniformly sourced, and that more uniform data sources could enhance the rigour of future research and the comparability of the data. Fourthly, we used Spearman's correlation analysis and regression analysis, meaning we looked at data from an ‘overall’ perspective and failed to fully consider the differences between individual drugs. Therefore, our results do not reflect the subtle differences between different drugs. This limitation can be remedied in the future through deeper subgroup analyses of indications.

## CONCLUSIONS

Based on our findings, we can provide several recommendations for the pricing policy of multi-indication anti-cancer drugs in China. First, when pricing anti-cancer drugs for solid tumours with multiple indications, China should link drug prices to the clinical efficacy of each indication and introduce a more transparent evaluation mechanism in the pricing process to ensure that patients can receive cost-effective treatments. We can learn from the approaches used in Japan and Taiwan, China where the evaluation of multi-indication drugs considers the clinical benefits, cost-effectiveness, and budget implications of different indications. This comprehensive assessment will determine the entry and payment standards for each indication, allowing for different prices for different indications. Second, we recommend that health insurance institutions improve the construction of databases to support the fine-grained measurement of health insurance, including real-world databases of drug efficacy and costs and disease epidemiology databases. From the experience of Taiwan, China, the tracking of real-world data also supports fine-grained management and control of costs in the later stages of health insurance. The establishment of databases can provide data support in the drug pricing process, making pricing more applicable to China's real-world situation. Furthermore, health insurance authorities should make full use of evidence from clinical trials, which provide information on clinical benefit, and further standardise and transparent clinical trial-based pharmacoeconomic evaluations. Finally, at the present stage in China, ‘pricing at one price’ remains a more mainstream and feasible approach for pharmaceutical pricing. If China intends to continue this strategy, it could draw lessons from the multi-year price management agreements implemented in countries such as the Netherlands and Belgium. Within the agreement period (*e.g.* four years), a dynamic pricing mechanism should be established to gradually incorporate evidence from new indications and coordinate pricing for comparable drugs, while balancing incentives for innovation and the sustainability of public health expenditures.

## Additional material


Online Supplementary Document

## References

[1] Kocarnik JM Compton K Dean FE Fu W Gaw BL Harvey JD Cancer Incidence, Mortality, Years of Life Lost, Years Lived With Disability, and Disability-Adjusted Life Years for 29 Cancer Groups From 2010 to 2019: A Systematic Analysis for the Global Burden of Disease Study 2019. JAMA Oncol. 2022;8:420–44. 10.1001/jamaoncol.2021.6987 34967848 PMC8719276

[2] Cao W Chen HD Yu YW Li N Chen WQ Changing profiles of cancer burden worldwide and in China: a secondary analysis of the global cancer statistics 2020. Chin Med J (Engl). 2021;134:783–91. 10.1097/CM9.0000000000001474 33734139 PMC8104205

[3] Qiu H Cao S Xu R Cancer incidence, mortality, and burden in China: a time-trend analysis and comparison with the United States and United Kingdom based on the global epidemiological data released in 2020. Cancer Commun (Lond). 2021;41:1037–48. 10.1002/cac2.12197 34288593 PMC8504144

[4] Zhao C Wang C Shen C Wang Q China’s achievements and challenges in improving health insurance coverage. Drug Discov Ther. 2018;12:1–6. 10.5582/ddt.2017.01064 29553080

[5] Yang Y Zhang Y Wagner AK Li H Shi L Guan X The impact of government reimbursement negotiation on targeted anticancer medicines use and cost in China: A cohort study based on national health insurance data. J Glob Health. 2023;13:04083. 10.7189/jogh.13.04083 37566690 PMC10420358

[6] Zhang Y Wushouer H Han S Fu M Guan X Shi L The impacts of government reimbursement negotiation on targeted anticancer medication price, volume and spending in China. BMJ Glob Health. 2021;6:e006196. 10.1136/bmjgh-2021-006196 34266848 PMC8286756

[7] Li K Liu H Jiang Q Overview and analysis on the national medical insurance negotiation drugs over the years: taking anti-cancer drugs as an example. Anti-Tumor Pharmacy. 2021;11:229–35.

[8] Molto C Hwang TJ Borrell M Andres M Gich I Barnadas A Clinical benefit and cost of breakthrough cancer drugs approved by the US Food and Drug Administration. Cancer. 2020;126:4390–9. 10.1002/cncr.33095 32697362

[9] Salas-Vega S Shearer E Mossialos E Relationship between costs and clinical benefits of new cancer medicines in Australia, France, the UK, and the US. Soc Sci Med. 2020;258:113042. 10.1016/j.socscimed.2020.113042 32480184

[10] Hofmarcher T Szilagyiova P Gustafsson A Dolezal T Rutkowski P Baxter C Access to novel cancer medicines in four countries in Central and Eastern Europe in relation to clinical benefit. ESMO Open. 2023;8:101593. 10.1016/j.esmoop.2023.101593 37413761 PMC10485399

[11] Mengato D Messori A Pricing of innovative drugs: correlation between incremental cost and survival gain in four countries. Ther Adv Med Oncol. 2016;8:309–11. 10.1177/1758834016644465 27482290 PMC4952021

[12] Center for drug evaluation, National Medical Product Administration China. [Information disclosure]. Available: https://www.cde.org.cn/main/xxgk/listpage/2f78f372d351c6851af7431c7710a731. Accessed: 11 April 2022. Chinese.

[13] Zhang Y Li Y Fu Q Han Z Wang D Umar SS Combined Immunotherapy and Targeted Therapies for Cancer Treatment: Recent Advances and Future Perspectives. Curr Cancer Drug Targets. 2023;23:251–64. 10.2174/1568009623666221020104603 36278447

[14] Liu J Pandya P Afshar S Therapeutic Advances in Oncology. Int J Mol Sci. 2021;22:2008. 33670524 10.3390/ijms22042008 PMC7922397

[15] Schnipper LE Davidson NE Wollins DS Tyne C Blayney DW Blum D American Society of Clinical Oncology Statement: A Conceptual Framework to Assess the Value of Cancer Treatment Options. J Clin Oncol. 2015;33:2563–77. 10.1200/JCO.2015.61.6706 26101248 PMC5015427

[16] Schnipper LE Davidson NE Wollins DS Blayney DW Dicker AP Ganz PA Updating the American Society of Clinical Oncology Value Framework: Revisions and Reflections in Response to Comments Received. J Clin Oncol. 2016;34:2925–34. 10.1200/JCO.2016.68.2518 27247218

[17] Cherny NI Dafni U Bogaerts J Latino NJ Pentheroudakis G Douillard JY ESMO-Magnitude of Clinical Benefit Scale version 1.1. Ann Oncol. 2017;28:2340–66. 10.1093/annonc/mdx310 28945867

[18] Cherny NI de Vries E Dafni U Garrett-Mayer E McKernin SE Piccart M Comparative Assessment of Clinical Benefit Using the ESMO-Magnitude of Clinical Benefit Scale Version 1.1 and the ASCO Value Framework Net Health Benefit Score. J Clin Oncol. 2019;37:336–49. 10.1200/JCO.18.00729 30707056

[19] National Health Commission of China. [Circular of the General Office of the National Health Commission on the Issuance of Guidelines for the Diagnosis and Treatment of Tumours and Blood Disease-Related Diseases (2022 Edition)]. 2022. Available: http://www.nhc.gov.cn/yzygj/s7659/202204/a0e67177df1f439898683e1333957c74.shtml. Accessed: 11 April 2022. Chinese.

[20] Gong J Su D Shang J Xu S Tang L Sun Z Cost-Effectiveness of Tislelizumab Versus Docetaxel for Previously Treated Advanced Non-Small-Cell Lung Cancer in China. Front Pharmacol. 2022;13:830380. 10.3389/fphar.2022.830380 35614942 PMC9124929

[21] Felip E Gridelli C Baas P Rosell R Stahel R Metastatic non-small-cell lung cancer: consensus on pathology and molecular tests, first-line, second-line, and third-line therapy: 1st ESMO Consensus Conference in Lung Cancer; Lugano 2010. Ann Oncol. 2011;22:1507–19. 10.1093/annonc/mdr150 21536661

[22] Yao X Ding J Liu Y Li P The New Drug Conditional Approval Process in China: Challenges and Opportunities. Clin Ther. 2017;39:1040–51. 10.1016/j.clinthera.2017.03.016 28431767

[23] Zou L Qi Y Jiang Y Tang L Du Y Zhao B Criteria and regulatory considerations for the conditional approval of innovative antitumor drugs in China: from the perspective of clinical reviewers. Cancer Commun (Lond). 2023;43:171–6. 10.1002/cac2.12400 36683350 PMC9926957

[24] Prasad V Mailankody S Research and Development Spending to Bring a Single Cancer Drug to Market and Revenues After Approval. JAMA Intern Med. 2017;177:1569–75. 10.1001/jamainternmed.2017.3601 28892524 PMC5710275

[25] DiMasi JA Grabowski HG Hansen RW Innovation in the pharmaceutical industry: New estimates of R&D costs. J Health Econ. 2016;47:20–33. 10.1016/j.jhealeco.2016.01.012 26928437

[26] Nirmalan PK Thomas R Sample sizes for clinical trials. Ophthalmology. 2007;114:615. 10.1016/j.ophtha.2006.11.002 17324699

[27] Julious SA Sample sizes for clinical trials with normal data. Stat Med. 2004;23:1921–86. 10.1002/sim.1783 15195324

[28] Manju MA Candel MJ Berger MP Optimal and maximin sample sizes for multicentre cost-effectiveness trials. Stat Methods Med Res. 2015;24:513–39. 10.1177/0962280215569293 25656551

[29] Lin S Huang Y Dong L Li M Wang Y Gu D The correlation between the costs and clinical benefits of PD-1/PD-L1 inhibitors in malignant tumors: An evaluation based on ASCO and ESMO frameworks. Front Pharmacol. 2023;14:1114304. 10.3389/fphar.2023.1114304 36909180 PMC9995671

[30] Luo J Ou S Wei H Qin X Peng R Wang S Value assessment of NMPA-approved new cancer drugs for solid cancer in China, 2016–2020. Front Public Health. 2023;11:1109668. 10.3389/fpubh.2023.1109668 36908440 PMC9998930

[31] Bao Y Liu Y Ma R Zhang P Li X The correlation between the costs and clinical benefits of national price-negotiated anticancer drugs for specific cancers in China. J Glob Health. 2023;13:04140. 10.7189/jogh.13.04140 37934965 PMC10629928

[32] Vokinger KN Hwang TJ Grischott T Reichert S Tibau A Rosemann T Prices and clinical benefit of cancer drugs in the USA and Europe: a cost-benefit analysis. Lancet Oncol. 2020;21:664–70. 10.1016/S1470-2045(20)30139-X 32359489

[33] Zhang Y Wei Y Li H Chen Y Guo Y Han S Prices and Clinical Benefit of National Price-Negotiated Anticancer Medicines in China. PharmacoEconomics. 2022;40:715-24. 10.1007/s40273-022-01161-7 35764914 PMC9270265

[34] Satoh E Sasaki Y Ohkuma R Takahashi T Kubota Y Ishida H Lack of correlation between the costs of anticancer drugs and clinical benefits in Japan. Cancer Sci. 2018;109:3896–901. 10.1111/cas.13831 30315613 PMC6272097

[35] Okabe A Hayashi H Maeda H Correlation of Anticancer Drug Prices with Outcomes of Overall Survival and Progression-Free Survival in Clinical Trials in Japan. Curr Oncol. 2023;30:1776–83. 10.3390/curroncol30020137 36826098 PMC9955512

