# Supplementary material for: Characteristics of indications, clinical trial evidence, clinical benefits and the costs of price-negotiated multi-indication drugs for solid tumours in China
Source: J Glob Health. 2025 May 9;15:04121. doi: 10.7189/jogh.15.04121 (PMC12061003; doi:10.7189/jogh.15.04121)
Supplement: Online Supplementary Document [file jogh-15-04121-s001.pdf]

Supplement to: Zhang P, Zhang MD, Ma R, Wei JX, Bao YW, Zhang LL, Qian XD, Su D, Li X. Characteristics of indications, clinical trial evidence, clinical benefits and the costs of price-negotiated multi-indication drugs for solid tumours in China. J Glob Health. 2025;15:04121.

Authors:
Pei Zhang[1], Mengdie Zhang[1] , Rui Ma[1] , Jingxuan Wei[1] , Yuwen Bao[2] , Lingli Zhang[3] , Xiaodan Qian[4] , Dan Su[4] , Xin Li[1,2]‡

[1]Department of Pharmaceutical Regulatory Science and Pharmacoeconomics, School of Pharmacy, Nanjing Medical University, Nanjing, China

[2]Department of Health Policy, School of Health Policy and Management, Nanjing Medical University, Nanjing, China

[3]School of International Pharmaceutical Business, China Pharmaceutical University, Nanjing, China

[4]Department of Pharmacy, The Second People's Hospital of Changzhou, the Third Affiliated Hospital of Nanjing Medical University, Changzhou, China

CORRESPONDENCE:
‡Xin Li, E-mail: xinli@njmu.edu.cn

STROBE Statement—checklist of items that should be included in reports of observational studies

	Item No	Recommendation	Page No
**Title and abstract**	1	(*a*) Indicate the study's design with a commonly used term in the title or the abstract	1-2
		(*b*) Provide in the abstract an informative and balanced summary of what was done and what was found	1-2
**Introduction**			
Background/rationale	2	Explain the scientific background and rationale for the investigation being reported	2-3
Objectives	3	State specific objectives, including any prespecified hypotheses	3
**Methods**			
Study design	4	Present key elements of study design early in the paper	3
Setting	5	Describe the setting, locations, and relevant dates, including periods of recruitment, exposure, follow-up, and data collection	3-5
Participants	6	(*a*) *Cohort study*—Give the eligibility criteria, and the sources and methods of selection of participants. Describe methods of follow-up<br>*Case-control study*—Give the eligibility criteria, and the sources and methods of case ascertainment and control selection. Give the rationale for the choice of cases and controls<br>*Cross-sectional study*—Give the eligibility criteria, and the sources and methods of selection of participants	4-5
		(*b*) *Cohort study*—For matched studies, give matching criteria and number of exposed and unexposed<br>*Case-control study*—For matched studies, give matching criteria and the number of controls per case	NA
Variables	7	Clearly define all outcomes, exposures, predictors, potential confounders, and effect modifiers. Give diagnostic criteria, if applicable	5-7
Data sources/ measurement	8*	For each variable of interest, give sources of data and details of methods of assessment (measurement). Describe comparability of assessment methods if there is more than one group	*5-7*
Bias	9	Describe any efforts to address potential sources of bias	4-5
Study size	10	Explain how the study size was arrived at	4-5
Quantitative variables	11	Explain how quantitative variables were handled in the analyses. If applicable, describe which groupings were chosen and why	7-8
Statistical methods	12	(*a*) Describe all statistical methods, including those used to control for confounding	7-8
		(*b*) Describe any methods used to examine subgroups and interactions	7-8
		(*c*) Explain how missing data were addressed	3
		(*d*) *Cohort study*—If applicable, explain how loss to follow-up was addressed<br>*Case-control study*—If applicable, explain how matching of cases and controls was addressed<br>*Cross-sectional study*—If applicable, describe analytical methods taking account of sampling strategy	N/A
		(*e*) Describe any sensitivity analyses	N/A

Continued on next page

**Results**			
Participants	13*	(a) Report numbers of individuals at each stage of study—eg numbers potentially eligible, examined for eligibility, confirmed eligible, included in the study, completing follow-up, and analysed	8
		(b) Give reasons for non-participation at each stage	4-5
		(c) Consider use of a flow diagram	Figure S1
Descriptive data	14*	(a) Give characteristics of study participants (eg demographic, clinical, social) and information on exposures and potential confounders	8-10
		(b) Indicate number of participants with missing data for each variable of interest	8-10
		(c) *Cohort study*—Summarise follow-up time (eg, average and total amount)	N/A
Outcome data	15*	*Cohort study*—Report numbers of outcome events or summary measures over time	8
		Case-control study—Report numbers in each exposure category, or summary measures of exposure	N/A
		Cross-sectional study—Report numbers of outcome events or summary measures	N/A
Main results	16	(*a*) Give unadjusted estimates and, if applicable, confounder-adjusted estimates and their precision (eg, 95% confidence interval). Make clear which confounders were adjusted for and why they were included	8-10
		(*b*) Report category boundaries when continuous variables were categorized	8-10
		(*c*) If relevant, consider translating estimates of relative risk into absolute risk for a meaningful time period	N/A
Other analyses	17	Report other analyses done—eg analyses of subgroups and interactions, and sensitivity analyses	N/A
**Discussion**			
Key results	18	Summarise key results with reference to study objectives	10
Limitations	19	Discuss limitations of the study, taking into account sources of potential bias or imprecision. Discuss both direction and magnitude of any potential bias	15-16
Interpretation	20	Give a cautious overall interpretation of results considering objectives, limitations, multiplicity of analyses, results from similar studies, and other relevant evidence	10-15
Generalisability	21	Discuss the generalisability (external validity) of the study results	14-15
**Other information**			
Funding	22	Give the source of funding and the role of the funders for the present study and, if applicable, for the original study on which the present article is based	16

*Give information separately for cases and controls in case-control studies and, if applicable, for exposed and unexposed groups in cohort and cross-sectional studies.

Note: An Explanation and Elaboration article discusses each checklist item and gives methodological background and published examples of transparent reporting. The STROBE checklist is best used in conjunction with this article (freely available on the Web sites of PLoS Medicine at http://www.plosmedicine.org/, Annals of Internal Medicine at http://www.annals.org/, and Epidemiology at http://www.epidem.com/). Information on the STROBE Initiative is available at www.strobe-statement.org.

Supplementary Figure 1: Flow chart for inclusion of multi-indication anticancer drugs. NRDL - National Reimbursement Drug List.

Figure S1

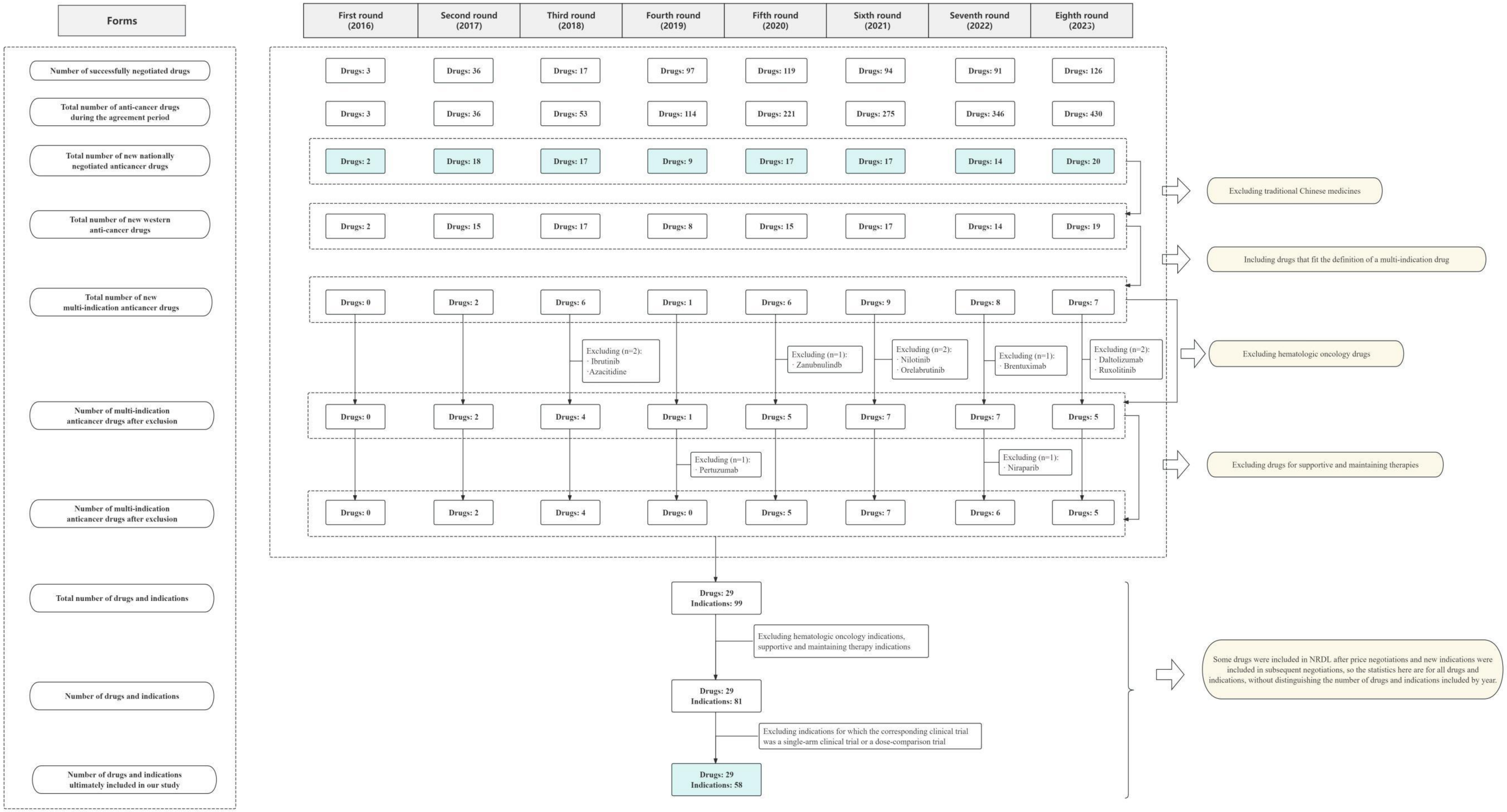

Supplementary table 1: Characteristics of muti-indication anticancer drugs

Serial number	Generic name	Origin	Whether it is an innovative drug (1 Yes, 2 No)	Number of indications	Mechanisms of action	Molecule types
1	Trastuzumab	Imported	2	3	Targeted therapy	Monoclonal antibody
2	Sorafenib	Imported	2	3	Targeted therapy	Small molecule
3	Pazopanib	Imported	2	3	Targeted therapy	Small molecule
4	Afatinib	Imported	2	2	Targeted therapy	Small molecule
5	Trametinib	Domestic	2	3	Targeted therapy	Small molecule
6	Dabrafenib	Domestic	2	3	Targeted therapy	Small molecule
7	Regorafenib	Imported	2	3	Targeted therapy	Small molecule
8	Furmonertinib	Domestic	2	2	Targeted therapy	Small molecule
9	Olaparib	Imported	2	3	Targeted therapy	Small molecule
10	Cetuximab	Imported	2	2	Targeted therapy	Monoclonal antibody
11	Sintilimab	Domestic	1	6	ICI	ICI
12	Tislelizumab	Domestic	1	11	ICI	ICI
13	Osimertinib	Imported	2	3	Targeted therapy	Small molecule
14	Anlotinib	Domestic	1	5	Targeted therapy	Small molecule
15	Apatinib	Domestic	2	3	Targeted therapy	Small molecule
16	Icotinib	Domestic	1	3	Targeted therapy	Small molecule
17	Almonertinib	Domestic	1	2	Targeted therapy	Small molecule
18	Camrelizumab	Domestic	1	9	ICI	ICI
19	Trastuzumab Emtansine	Imported	2	2	Targeted therapy	ADC
20	Toripalimab	Domestic	1	6	ICI	ICI
21	Pyrotinib	Domestic	1	2	Targeted therapy	Small molecule
22	Donafenib	Domestic	1	2	Targeted therapy	Small molecule
23	Abemaciclib	Imported	2	3	Targeted therapy	Small molecule
24	Dalpiciclib	Domestic	1	3	Targeted therapy	Small molecule

ICI – immune checkpoint inhibitor, ADC – antibody-drug conjugates.

Supplementary table 2: Characteristics of indications for muti-indication anticancer drugs

Serial number	Generic name	Indication	Lines of therapy	Year of initial NRDL price negotiation	Time to market	Time to enroll in NRDL	Delay between marketing and enrolling in NRDL	Original indication / Supplemental indication	Type of approval for indications	Type of treatment
1	Trastuzumab	BRCA	First-line	2017	September-02	January-18	15.33	Original	Priority Approval	Combination therapy
		STAD	First-line	2019	September-02	January-20	17.33	Original	Priority Approval	Combination therapy
2	Sorafenib	RCC	First-line	2017	September-06	January-18	11.34	Original	Normal Approval	Monotherapy
		HCC	First-line	2017	June-08	January-18	9.59	Supplemental	Normal Approval	Monotherapy
		STAD	Second-line	2017	March-17	January-18	0.84	Supplemental	Priority Approval	Monotherapy
3	Pazopanib	RCC	First-line	2018	February-17	January-19	1.92	Original	Normal Approval	Monotherapy
		RCC	Second-line	2018	February-17	January-19	1.92	Original	Normal Approval	Monotherapy
4	Afatinib	NSCLC	First-line	2018	February-17	January-19	1.92	Original	Special Approval	Monotherapy
		SQ-NSCLC	Second-line	2018	February-17	January-19	1.92	Original	Special Approval	Monotherapy
5	Trametinib	SKCM	First-line	2020	December-19	January-21	1.09	Original	Priority Approval	Combination therapy
6	Dabrafenib	SKCM	First-line	2020	December-19	January-21	1.09	Original	Priority Approval	Combination therapy
7	Regorafenib	HCC	Second-line	2021	December-17	January-22	4.09	Supplemental	Priority Approval	Monotherapy
		CRC	Third-line	2021	May-17	January-22	4.67	Original	Special Approval	Monotherapy
8	Furmonertinib	NSCLC	First-line	2021	June-22	January-23	0.67	Supplemental	Conditional Approval	Monotherapy
9	Olaparib	PRAD	Second-line	2021	June-21	January-23	1.60	Supplemental	Conditional Approval	Monotherapy
10	Cetuximab	CRC	First-line	2021	December-05	January-22	16.10	Original	Normal Approval	Combination therapy
		HNSC	First-line	2021	March-20	January-22	1.84	Supplemental	Priority Approval	Combination therapy
11	Sintilimab	NSQ-NSCLC	First-line	2021	February-21	January-22	0.92	Supplemental	Normal Approval	Combination therapy
		SQ-NSCLC	First-line	2021	June-21	January-22	0.59	Supplemental	Normal Approval	Combination therapy
		HCC	First-line	2021	June-21	January-22	0.59	Supplemental	Priority Approval	Combination therapy
		ESCC	First-line	2022	June-22	January-23	0.61	Supplemental	Normal Approval	Combination therapy
		GC/GEJC	First-line	2022	June-22	January-23	0.61	Supplemental	Normal Approval	Combination therapy
12	Tislelizumab	SQ-NSCLC	First-line	2021	January-20	January-22	2.00	Supplemental	Normal Approval	Combination therapy
		NSQ-NSCLC	First-line	2022	June-21	January-23	1.62	Supplemental	Normal Approval	Combination therapy
		NSCLC	Second-line	2022	June-21	January-23	1.63	Supplemental	Normal Approval	Monotherapy
		ESCC	Second-line	2022	April-22	January-23	0.80	Supplemental	Normal Approval	Monotherapy
		NPC	First-line	2022	June-22	January-23	0.64	Supplemental	Normal Approval	Combination therapy
		GC/GEJC	First-line	2023	February-23	January-24	0.92	Supplemental	Normal Approval	Combination therapy
		ESCC	First-line	2023	May-23	January-24	0.67	Supplemental	Normal Approval	Monotherapy

Serial number	Generic name	Indication	Lines of therapy	Year of initial NRDL price negotiation	Time to market	Time to enroll in NRDL	Delay between marketing and enrolling in NRDL	Original indication / Supplemental indication	Type of approval for indications	Type of treatment
13	Osimertinib	NSCLC	First-line	2021	August-19	January-22	2.42	Supplemental	Priority Approval	Monotherapy
		NSCLC	Second-line	2021	March-17	January-22	4.84	Original	Special Approval	Monotherapy
14	Anlotinib	NSCLC	Third-line	2021	May-18	January-22	3.67	Original	Special Approval	Monotherapy
		SCLC	Third-line	2021	August-19	January-22	2.42	Supplemental	Conditional Approval	Monotherapy
		MTC	First-line	2021	February-21	January-22	0.92	Supplemental	Conditional Approval	Monotherapy
		THCA	Second-line	2023	April-22	January-24	1.75	Supplemental	Normal Approval	Monotherapy
15	Apatinib	GC/GEJC	Third-line	2021	October-14	January-22	7.26	Original	Normal Approval	Monotherapy
		HCC	Second-line	2021	December-20	January-22	1.08	Supplemental	Normal Approval	Monotherapy
		HCC	First-line	2023	January-23	January-24	1.00	Supplemental	Normal Approval	Combination therapy
16	Icotinib	NSCLC	First-line	2021	November-14	January-22	7.17	Supplemental	Normal Approval	Monotherapy
		NSCLC	Third-line	2021	June-11	January-22	10.59	Original	Normal Approval	Monotherapy
17	Almonertinib	NSCLC	First-line	2022	December-21	January-23	1.10	Supplemental	Priority Approval	Monotherapy
18	Camrelizumab	NSQ-NSCLC	First-line	2022	June-20	January-23	2.65	Supplemental	Special Approval	Combination therapy
		ESCC	Second-line	2022	June-20	January-23	2.66	Supplemental	Special Approval	Monotherapy
		NPC	First-line	2022	June-21	January-23	1.66	Supplemental	Priority Approval	Combination therapy
		ESCC	First-line	2022	December-21	January-23	1.16	Supplemental	Normal Approval	Combination therapy
		SQ-NSCLC	First-line	2022	December-21	January-23	1.17	Supplemental	Normal Approval	Combination therapy
		HCC	First-line	2023	March-20	January-24	3.84	Supplemental	Normal Approval	Combination therapy
19	Trastuzumab Emtansine	BRCA	Second-line	2022	June-21	January-23	1.59	Supplemental	Priority Approval	Monotherapy
20	Toripalimab	NPC	First-line	2023	November-21	January-24	2.17	Supplemental	Normal Approval	Combination therapy
		ESCC	First-line	2023	May-22	January-24	1.67	Supplemental	Normal Approval	Combination therapy
		NSQ-NSCLC	First-line	2023	September-22	January-24	1.33	Supplemental	Normal Approval	Combination therapy
21	Pyrotinib	BRCA	Second-line	2023	July-20	January-24	3.50	Supplemental	Special Approval	Combination therapy
22	Donafenib	HCC	First-line	2023	June-21	January-24	2.59	Original	Priority Approval	Monotherapy
		THCA	Second-line	2023	August-22	January-24	1.42	Supplemental	Normal Approval	Monotherapy
23	Abemaciclib	BRCA	First-line	2023	December-20	January-24	3.08	Original	Normal Approval	Combination therapy
		BRCA	Second-line	2023	December-20	January-24	3.08	Original	Normal Approval	Combination therapy
24	Dalpiciclib	BRCA	First-line	2023	June-23	January-24	0.59	Supplemental	Normal Approval	Combination therapy
		BRCA	Second-line	2023	December-21	January-24	2.08	Original	Special Approval	Combination therapy

BRCA – Breast Cancer, STCA – Stomach Cancer, RCC – Renal cell carcinoma, HCC – Hepatocellular carcinoma, NSCLC – Non-small-cell lung cancer, SQ-NSCLC – Squamous non-small-cell lung cancer, SKCM – Skin Cutaneous Melanoma, CRC – Colorectal cancer, PRAD – Prostate adenocarcinoma, HNSCC – Head and Neck squamous cell carcinoma, NSQ-NSCLC – Non-squamous non-small-cell lung cancer, ESCC – Esophageal

squamous cell carcinoma, GC/GEJC – Gastric or gastroesophageal junction adenocarcinoma, SCLC – Small cell lung cancer, MTC – Medullary thyroid cancer, THCA – Thyroid Cancer, NPC – Nasopharyngeal carcinoma, NRDL – National Reimbursement Drug List.

Supplementary table 3: Clinical trials evidence

Serial number	Generic name	Origin	Indication	Lines of therapy	NCT	Reference	Phase of Clinical Trials	Number of patients enrolled	Blinding
1	Trastuzumab	Imported	BRCA	First-line	NCT05621434	(1)	III	469	Open label
			STAD	First-line	NCT01041404	(2)	III	584	Open label
2	Sorafenib	Imported	RCC	First-line	NCT00073307	(3)	III	903	Double-blind
			HCC	First-line	NCT00105443	(4)	III	602	Double-blind
			STAD	Second-line	NCT00984282	(5)	III	417	Double-blind
3	Pazopanib	Imported	RCC	First-line	NCT00720941	(6)	III	1110	Open label
			RCC	Second-line	NCT00334282	(7)	III	435	Double-blind
4	Afatinib	Imported	NSCLC	First-line	NCT00949650	(8)	III	345	Open label
			SQ-NSCLC	Second-line	NCT01523587	(9)	III	795	Open label
5	Trametinib	Domestic	SKCM	First-line	NCT01584648	(10)	III	423	Double-blind
6	Dabrafenib	Domestic	SKCM	First-line	NCT01597908	(11)	III	704	Open label
7	Regorafenib	Imported	HCC	Second-line	NCT01774344	(12)	III	573	Double-blind
			CRC	Third-line	NCT01103323	(13)	III	760	Double-blind
8	Furmonertinib	Domestic	NSCLC	First-line	NCT03787992	(14)	III	358	Double-blind
9	Olaparib	Imported	PRAD	Second-line	NCT02987543	(15)	III	245	Open label
10	Cetuximab	Imported	CRC	First-line	NCT01228734	(16)	III	393	Open label
			HNSC	First-line	NCT00122460	(17)	III	442	Open label
11	Sintilimab	Domestic	NSQ-NSCLC	First-line	NCT03607539 （ORIENT-11）	(18)	III	397	Double-blind
			SQ-NSCLC	First-line	NCT03607539 （ORIENT-12）	(19)	III	357	Double-blind
			HCC	First-line	NCT03794440	(20)	II/III	595	Open label
			ESCC	First-line	NCT03748134	(21)	III	659	Double-blind
			GC/GEJC	First-line	NCT03745170	(22)	III	650	Double-blind
12	Tislelizumab	Domestic	SQ-NSCLC	First-line	NCT03594747	(23)	III	360	Open label
			NSQ-NSCLC	First-line	NCT03663205	(24)	III	332	Open label
			NSCLC	Second-line	NCT03358875	(25)	III	805	Open label
			ESCC	Second-line	NCT03430843	(26)	III	512	Open label
			NPC	First-line	NCT03924986	(27)	III	263	Double-blind
			GC/GEJC	First-line	NCT03777657	(28)	III	546	Double-blind

Serial number	Generic name	Origin	Indication	Lines of therapy	NCT	Reference	Phase of Clinical Trials	Number of patients enrolled	Blinding
			ESCC	First-line	NCT03783442	(29)	III	649	Double-blind
13	Osimertinib	Imported	NSCLC	First-line	NCT02296125	(30)	III	556	Double-blind
			NSCLC	Second-line	NCT02151981	(31)	III	419	Open label
14	Anlotinib	Domestic	NSCLC	Third-line	NCT02388919	(32)	III	437	Double-blind
			SCLC	Third-line	NCT03059797	(33)	II/III	120	Double-blind
			MTC	First-line	NCT02586350	(34)	II/III	91	Double-blind
			THCA	Second-line	NCT02586337	(35)	II/III	113	Double-blind
15	Apatinib	Domestic	GC/GEJC	Third-line	NCT01512745	(36)	III	267	Double-blind
			HCC	Second-line	NCT02329860	(37)	III	400	Double-blind
			HCC	First-line	NCT03764293	(38)	III	543	Open label
16	Icotinib	Domestic	NSCLC	First-line	NCT01719536	(39)	III	285	Open label
			NSCLC	Third-line	NCT01040780	(40)	III	395	Double-blind
17	Almonertinib	Domestic	NSCLC	First-line	NCT03849768	(41)	II/III	429	Double-blind
18	Camrelizumab	Domestic	NSQ-NSCLC	First-line	NCT03134872	(42)	III	412	Open label
			ESCC	Second-line	NCT03099382	(43)	III	457	Open label
			NPC	First-line	NCT03707509	(44)	III	263	Double-blind
			ESCC	First-line	NCT03691090	(45)	III	596	Double-blind
			SQ-NSCLC	First-line	NCT03668496	(46)	III	389	Double-blind
			HCC	First-line	NCT03764293	(47)	III	543	Double-blind
19	Trastuzumab Emtansine	Imported	BRCA	Second-line	NCT00829166	(48)	III	991	Open label
20	Toripalimab	Domestic	NPC	First-line	NCT03581786	(49)	III	289	Double-blind
			ESCC	First-line	NCT03829969	(50)	III	514	Double-blind
			NSQ-NSCLC	First-line	NCT03856411	(51)	III	245	Double-blind
21	Pyrotinib	Domestic	BRCA	Second-line	NCT03080805	(52)	III	266	Open label
22	Donafenib	Domestic	HCC	First-line	NCT02645981	(53)	II/III	659	Open label
			THCA	Second-line	NCT03602495	(54)	III	659	Double-blind
23	Abemaciclib	Imported	BRCA	First-line	NCT02246621	(55)	III	493	Double-blind
			BRCA	Second-line	NCT02107703	(56)	III	669	Double-blind
24	Dalpiciclib	Domestic	BRCA	First-line	NCT03966898	(57)	III	672	Double-blind
			BRCA	Second-line	NCT03927456	(58)	III	456	Double-blind

BRCA – Breast Cancer, STCA – Stomach Cancer, RCC – Renal cell carcinoma, HCC – Hepatocellular carcinoma, NSCLC – Non-small-cell lung cancer, SQ-NSCLC – Squamous non-small-cell lung cancer, SKCM – Skin Cutaneous Melanoma, CRC – Colorectal cancer, PRAD – Prostate adenocarcinoma, HNSCC – Head and Neck squamous cell carcinoma, NSQ-NSCLC – Non-squamous non-small-cell lung cancer, ESCC – Esophageal

squamous cell carcinoma, GC/GEJC – Gastric or gastroesophageal junction adenocarcinoma, SCLC – Small cell lung cancer, MTC – Medullary thyroid cancer, THCA – Thyroid Cancer, NPC – Nasopharyngeal carcinoma, NCT – Number of clinical trial.

Supplementary table 4: Clinical benefits

Serial number	Generic name	Indication	Lines of therapy	Evaluation index	ΔPFS%	ΔOS%	ASCO-VF scores	ESMO-MCBS scores
1	Trastuzumab	BRCA	First-line	OS	60.00%	23.65%	33.65	4
		STAD	First-line	OS	21.82%	24.32%	26.00	3
2	Sorafenib	RCC	First-line	OS	66.67%	17.11%	28.00	3
		HCC	First-line	OS	-16.33%	35.44%	27.67	3
		STAD	Second-line	PFS	86.21%	/	56.80	2
3	Pazopanib	RCC	First-line	OS	-11.58%	-2.75%	28.50	4
		RCC	Second-line	OS	119.05%	11.71%	25.00	3
4	Afatinib	NSCLC	First-line	PFS	60.87%	0.00%	47.77	4
		SQ-NSCLC	Second-line	OS	36.84%	16.18%	29.00	2
5	Trametinib	SKCM	First-line	PFS	5.68%	34.22%	29.00	4
6	Dabrafenib	SKCM	First-line	OS	56.16%	/	41.00	5
7	Regorafenib	HCC	Second-line	OS	106.67%	35.90%	53.00	4
		CRC	Third-line	OS	11.76%	28.00%	22.60	1
8	Furmonertinib	NSCLC	First-line	PFS	87.39%	/	54.55	3
9	Olaparib	PRAD	Second-line	OS	105.56%	22.52%	22.60	3
10	Cetuximab	CRC	First-line	PFS	24.32%	26.06%	24.00	4
		HNSC	First-line	OS	69.70%	36.49%	30.30	3
11	Sintilimab	NSQ-NSCLC	First-line	OS	78.00%	44.05%	52.29	5
		SQ-NSCLC	First-line	PFS	4.08%	/	30.32	4
		HCC	First-line	PFS	64.29%	/	49.26	5
		ESCC	First-line	OS	26.32%	33.60%	47.20	4
		GC/GEJC	First-line	OS	24.56%	23.58%	23.40	2
12	Tislelizumab	SQ-NSCLC	First-line	PFS	38.18%	/	52.69	**4**
		NSQ-NSCLC	First-line	PFS	27.63%	/	28.40	4
		NSCLC	Second-line	OS	24.24%	21.71%	25.00	3
		ESCC	Second-line	OS	-23.81%	36.51%	30.00	4
		NPC	First-line	PFS	24.32%	/	38.40	4
		GC/GEJC	First-line	OS	22.03%	36.51%	36.00	3
		ESCC	First-line	OS	30.36%	62.26%	54.00	3
13	Osimertinib	NSCLC	First-line	OS	85.29%	21.38%	20.29	3
		NSCLC	Second-line	OS	129.55%	19.11%	23.00	4

Serial number	Generic name	Indication	Lines of therapy	Evaluation index	ΔPFS%	ΔOS%	ASCO-VF scores	ESMO-MCBS scores
14	Anlotinib	NSCLC	Third-line	OS	285.71%	52.38%	41.33	4
		SCLC	Third-line	OS	485.71%	48.98%	63.20	4
		MTC	First-line	PFS	86.49%	/	46.93	5
		THCA	Second-line	PFS	382.14%	/	79.20	5
15	Apatinib	GC/GEJC	Third-line	OS	44.44%	38.30%	29.10	3
		HCC	Second-line	OS	136.84%	27.94%	37.50	2
		HCC	First-line	OS	51.35%	45.39%	56.36	5
16	Icotinib	NSCLC	First-line	PFS	41.77%	-4.98%	53.02	4
		NSCLC	Third-line	OS	35.29%	-4.32%	12.80	2
17	Almonertinib	NSCLC	First-line	PFS	94.95%	/	40.98	3
18	Camrelizumab	NSQ-NSCLC	First-line	OS	36.14%	36.10%	37.26	4
		ESCC	Second-line	OS	0.00%	32.69%	50.93	2
		NPC	First-line	PFS	40.58%	/	36.80	3
		ESCC	First-line	OS	23.21%	27.50%	29.70	3
		SQ-NSCLC	First-line	PFS	73.47%	/	50.48	5
		HCC	First-line	OS	51.35%	45.39%	52.36	5
19	Trastuzumab Emtansine	BRCA	Second-line	OS	50.00%	23.11%	41.80	4
20	Toripalimab	NPC	First-line	PFS	46.25%	/	38.40	3
		ESCC	First-line	OS	3.64%	54.55%	42.00	5
		NSQ-NSCLC	First-line	PFS	176.36%	/	57.60	5
21	Pyrotinib	BRCA	Second-line	PFS	83.82%	/	47.26	5
22		HCC	First-line	OS	2.78%	17.48%	16.90	2
	Donafenib	THCA	Second-line	PFS	101.56%	/	58.21	5
23	Abemaciclib	BRCA	First-line	PFS	90.92%	/	34.58	3
		BRCA	Second-line	OS	76.34%	25.20%	37.44	4
24	Dalpiciclib	BRCA	First-line	PFS	83.08%	/	49.78	5
		BRCA	Second-line	PFS	68.13%	/	55.20	5

BRCA – Breast Cancer, STCA – Stomach Cancer, RCC – Renal cell carcinoma, HCC – Hepatocellular carcinoma, NSCLC – Non-small-cell lung cancer, SQ-NSCLC – Squamous non-small-cell lung cancer, SKCM – Skin Cutaneous Melanoma, CRC – Colorectal cancer, PRAD – Prostate adenocarcinoma, HNSCC – Head and Neck squamous cell carcinoma, NSQ-NSCLC – Non-squamous non-small-cell lung cancer, ESCC – Esophageal squamous cell carcinoma, GC/GEJC – Gastric or gastroesophageal junction adenocarcinoma, SCLC – Small cell lung cancer, MTC – Medullary thyroid cancer, THCA – Thyroid Cancer, NPC – Nasopharyngeal carcinoma, ΔPFS% – percentage improvement of progression-free survival, ΔOS% – percentage improvement of overall survival, ASCO-VF scores – American Society of Clinical Oncology-Value Framework scores, ESMO-MCBS scores – European Society for Medical Oncology-Magnitude of Clinical Benefit Scale scores.

Supplementary table 5: Quantitative assignment of indication characteristics, clinical trial evidence, clinical benefits, and epidemiology

Variable			Assignment
Characteristics of indications	X_1	Delay between marketing and enrolling in NRDL (year)	Inclusion of original value
	X_2	Lines of therapy	First line = 1, second line = 2, third line = 3
	X_3	Type of marketing approval	Regular approval = 1, Priority review and approval = 2, Special Approval = 3, Conditional Approval = 4
	X_4	Type of therapy	Monotherapy = 1, Combination therapy = 2
Clinical trial evidences	X_5	Number of patients enrolled	Inclusion of original value
	X_6	Phase of Clinical Trials	Phase II = 1, Phase II/III = 2, Phase III = 3
	X_7	Blindness	Double-blind = 1, open label = 2
Clinical benefits	X_8	△OS%	Inclusion of original value
	X_9	△PFS%	Inclusion of original value
	X_{10}	ACSO-VF score	Inclusion of original value
	X_{11}	ESMO-MCBS score	Inclusion of original value

NRDL – National Reimbursement Drug List, ΔPFS% – percentage improvement of progression-free survival, ΔOS% – percentage improvement of overall survival, ASCO-VF scores – American Society of Clinical Oncology-Value Framework scores, ESMO-MCBS scores – European Society for Medical Oncology-Magnitude of Clinical Benefit Scale scores.

References:

1. Slamon DJ, Leyland-Jones B, Shak S, Fuchs H, Paton V, Bajamonde A*, et al.*. Use of chemotherapy plus a monoclonal antibody against HER2 for metastatic breast cancer that overexpresses HER2. N Engl J Med 2001;344(11):783-92.
2. Bang YJ, Van Cutsem E, Feyereislova A, Chung HC, Shen L, Sawaki A*, et al.*. Trastuzumab in combination with chemotherapy versus chemotherapy alone for treatment of HER2-positive advanced gastric or gastro-oesophageal junction cancer (ToGA): a phase 3, open-label, randomised controlled trial. Lancet 2010;376(9742):687-97.
3. Escudier B, Eisen T, Stadler WM, Szczylik C, Oudard S, Staehler M*, et al.*. Sorafenib for treatment of renal cell carcinoma: Final efficacy and safety results of the phase III treatment approaches in renal cancer global evaluation trial. J Clin Oncol 2009;27(20):3312-8.
4. Llovet JM, Ricci S, Mazzaferro V, Hilgard P, Gane E, Blanc JF*, et al.*. Sorafenib in advanced hepatocellular carcinoma. N Engl J Med 2008;359(4):378-90.
5. Brose MS, Nutting CM, Jarzab B, Elisei R, Siena S, Bastholt L*, et al.*. Sorafenib in radioactive iodine-refractory, locally advanced or metastatic differentiated thyroid cancer: a randomised, double-blind, phase 3 trial. Lancet 2014;384(9940):319-28.
6. Motzer RJ, Hutson TE, Cella D, Reeves J, Hawkins R, Guo J*, et al.*. Pazopanib versus sunitinib in metastatic renal-cell carcinoma. N Engl J Med 2013;369(8):722-31.
7. Motzer RJ, Hutson TE, Cella D, Reeves J, Hawkins R, Guo J*, et al.*. Pazopanib versus sunitinib in metastatic renal-cell carcinoma. N Engl J Med 2013;369(8):722-31.
8. Sequist LV, Yang JC, Yamamoto N, O'Byrne K, Hirsh V, Mok T*, et al.*. Phase III study of afatinib or cisplatin plus pemetrexed in patients with metastatic lung adenocarcinoma with EGFR mutations. Journal of clinical oncology 2013;31(27):3327-3334.
9. Soria JC, Felip E, Cobo M, Lu S, Syrigos K, Lee KH*, et al.*. Afatinib versus erlotinib as second-line treatment of patients with advanced squamous cell carcinoma of the lung (LUX-Lung 8): an open-label randomised controlled phase 3 trial. Lancet Oncol 2015;16(8):897-907.
10. Long GV, Stroyakovskiy D, Gogas H, Levchenko E, de Braud F, Larkin J*, et al.*. Dabrafenib and trametinib versus dabrafenib and placebo for Val600 BRAF-mutant melanoma: a multicentre, double-blind, phase 3 randomised controlled trial. Lancet 2015;386(9992):444-51.
11. Grob JJ, Amonkar MM, Karaszewska B, Schachter J, Dummer R, Mackiewicz A*, et al.*. Comparison of dabrafenib and trametinib combination therapy with vemurafenib monotherapy on health-related quality of life in patients with unresectable or metastatic cutaneous BRAF Val600-mutation-positive melanoma (COMBI-v): results of a phase 3, open-label, randomised trial. Lancet Oncol 2015;16(13):1389-98.
12. Bruix J, Qin S, Merle P, Granito A, Huang YH, Bodoky G*, et al.*. Regorafenib for patients with hepatocellular carcinoma who progressed on sorafenib treatment (RESORCE): a randomised, double-blind, placebo-controlled, phase 3 trial. Lancet 2017;389(10064):56-66.
13. Grothey A, Van Cutsem E, Sobrero A, Siena S, Falcone A, Ychou M*, et al.*. Regorafenib monotherapy for previously treated metastatic colorectal cancer (CORRECT): an international, multicentre, randomised, placebo-controlled, phase 3 trial. Lancet 2013;381(9863):303-12.
14. Shi Y, Chen G, Wang X, Liu Y, Wu L, Hao Y*, et al.*. Furmonertinib (AST2818) versus gefitinib as

first-line therapy for Chinese patients with locally advanced or metastatic EGFR mutation-positive non-small-cell lung cancer (FURLONG): a multicentre, double-blind, randomised phase 3 study. The Lancet Respiratory Medicine 2022;10(11):1019-1028.
15. de Bono J, Mateo J, Fizazi K, Saad F, Shore N, Sandhu S, *et al.*. Olaparib for Metastatic Castration-Resistant Prostate Cancer. N Engl J Med 2020;382(22):2091-2102.
16. Qin S, Li J, Wang L, Xu J, Cheng Y, Bai Y, *et al.*. Efficacy and Tolerability of First-Line Cetuximab Plus Leucovorin, Fluorouracil, and Oxaliplatin (FOLFOX-4) Versus FOLFOX-4 in Patients With RAS Wild-Type Metastatic Colorectal Cancer: The Open-Label, Randomized, Phase III TAILOR Trial. J Clin Oncol 2018;36(30):3031-3039.
17. Vermorken JB, Mesia R, Rivera F, Remenar E, Kawecki A, Rottey S, *et al.*. Platinum-based chemotherapy plus cetuximab in head and neck cancer. N Engl J Med 2008;359(11):1116-27.
18. Yang Y, Wang Z, Fang J, Yu Q, Han B, Cang S, *et al.*. Efficacy and Safety of Sintilimab Plus Pemetrexed and Platinum as First-Line Treatment for Locally Advanced or Metastatic Nonsquamous NSCLC: a Randomized, Double-Blind, Phase 3 Study (Oncology pRogram by InnovENT anti-PD-1-11). J Thorac Oncol 2020;15(10):1636-1646.
19. Zhou C, Wu L, Fan Y, Wang Z, Liu L, Chen G, *et al.*. Sintilimab Plus Platinum and Gemcitabine as First-Line Treatment for Advanced or Metastatic Squamous NSCLC: Results From a Randomized, Double-Blind, Phase 3 Trial (ORIENT-12). J Thorac Oncol 2021;16(9):1501-1511.
20. Ren Z, Xu J, Bai Y, Xu A, Cang S, Du C, *et al.*. Sintilimab plus a bevacizumab biosimilar (IBI305) versus sorafenib in unresectable hepatocellular carcinoma (ORIENT-32): a randomised, open-label, phase 2-3 study. Lancet Oncol 2021;22(7):977-990.
21. Lu Z, Wang J, Shu Y, Liu L, Kong L, Yang L, *et al.*. Sintilimab versus placebo in combination with chemotherapy as first line treatment for locally advanced or metastatic oesophageal squamous cell carcinoma (ORIENT-15): multicentre, randomised, double blind, phase 3 trial. BMJ 2022;377:e068714.
22. Xu J, Jiang H, Pan Y, Gu K, Cang S, Han L, *et al.*. Sintilimab Plus Chemotherapy for Unresectable Gastric or Gastroesophageal Junction Cancer: The ORIENT-16 Randomized Clinical Trial. JAMA 2023;330(21):2064-2074.
23. Wang J, Lu S, Yu X, Hu Y, Sun Y, Wang Z, *et al.*. Tislelizumab plus chemotherapy vs chemotherapy alone as first-line treatment for advanced squamous non ‑ small-cell lung cancer: a phase 3 randomized clinical trial. JAMA oncology 2021;7(5):709-717.
24. Lu S, Wang J, Yu Y, Yu X, Hu Y, Ai X, *et al.*. Tislelizumab Plus Chemotherapy as First-Line Treatment for Locally Advanced or Metastatic Nonsquamous NSCLC (RATIONALE 304): A Randomized Phase 3 Trial. J Thorac Oncol 2021;16(9):1512-1522.
25. Zhou C, Huang D, Fan Y, Yu X, Liu Y, Shu Y, *et al.*. Tislelizumab Versus Docetaxel in Patients With Previously Treated Advanced NSCLC (RATIONALE-303): A Phase 3, Open-Label, Randomized Controlled Trial. J Thorac Oncol 2023;18(1):93-105.
26. Shen L, Kato K, Kim SB, Ajani JA, Zhao K, He Z, *et al.*. Tislelizumab Versus Chemotherapy as Second-Line Treatment for Advanced or Metastatic Esophageal Squamous Cell Carcinoma (RATIONALE-302): A Randomized Phase III Study. J Clin Oncol 2022;40(26):3065-3076.
27. Yang Y, Pan J, Wang H, Zhao Y, Qu S, Chen N, *et al.*. Tislelizumab plus chemotherapy as first-line treatment for recurrent or metastatic nasopharyngeal cancer: A multicenter phase 3 trial (RATIONALE-309). Cancer Cell 2023;41(6):1061-1072.e4.

28. Moehler MH, Kato K, Arkenau H, Oh D, Tabernero J, Cruz-Correa M, *et al.*. Rationale 305: Phase 3 study of tislelizumab plus chemotherapy vs placebo plus chemotherapy as first-line treatment (1L) of advanced gastric or gastroesophageal junction adenocarcinoma (GC/GEJC). In: American Society of Clinical Oncology; 2023.
29. Xu J, Kato K, Raymond E, Hubner RA, Shu Y, Pan Y, *et al.*. Tislelizumab plus chemotherapy versus placebo plus chemotherapy as first-line treatment for advanced or metastatic oesophageal squamous cell carcinoma (RATIONALE-306): a global, randomised, placebo-controlled, phase 3 study. Lancet Oncol 2023;24(5):483-495.
30. Soria J, Ohe Y, Vansteenkiste J, Reungwetwattana T, Chewaskulyong B, Lee KH, *et al.*. Osimertinib in untreated EGFR-mutated advanced non－small-cell lung cancer. New England journal of medicine 2018;378(2):113-125.
31. Mok TS, Wu Y, Ahn M, Garassino MC, Kim HR, Ramalingam SS, *et al.*. Osimertinib or Platinum-Pemetrexed in EGFR T790M-Positive Lung Cancer. N Engl J Med 2017;376(7):629-640.
32. Zhou M, Chen X, Zhang H, Xia L, Tong X, Zou L, *et al.*. China National Medical Products Administration approval summary: anlotinib for the treatment of advanced non-small cell lung cancer after two lines of chemotherapy. Cancer Commun (Lond) 2019;39(1):36.
33. Cheng Y, Wang Q, Li K, Shi J, Liu Y, Wu L, *et al.*. Anlotinib vs placebo as third- or further-line treatment for patients with small cell lung cancer: a randomised, double-blind, placebo-controlled Phase 2 study. Br J Cancer 2021;125(3):366-371.
34. Li D, Chi Y, Chen X, Ge M, Zhang Y, Guo Z, *et al.*. Anlotinib in Locally Advanced or Metastatic Medullary Thyroid Carcinoma: A Randomized, Double-Blind Phase IIB Trial. Clin Cancer Res 2021;27(13):3567-3575.
35. Chi Y, Zheng X, Zhang Y, Shi F, Cheng Y, Guo Z, *et al.*. Anlotinib in Locally Advanced or Metastatic Radioiodine-Refractory Differentiated Thyroid Carcinoma: A Randomized, Double-Blind, Multicenter Phase II Trial. Clin Cancer Res 2023;29(20):4047-4056.
36. Li J, Qin S, Xu J, Xiong J, Wu C, Bai Y, *et al.*. Randomized, double-blind, placebo-controlled phase III trial of apatinib in patients with chemotherapy-refractory advanced or metastatic adenocarcinoma of the stomach or gastroesophageal junction. Journal of Clinical Oncology 2016;34(13):1448-1454.
37. Qin S, Li Q, Gu S, Chen X, Lin L, Wang Z, *et al.*. Apatinib as second-line or later therapy in patients with advanced hepatocellular carcinoma (AHELP): a multicentre, double-blind, randomised, placebo-controlled, phase 3 trial. Lancet Gastroenterol Hepatol 2021;6(7):559-568.
38. Qin S, Chan SL, Gu S, Bai Y, Ren Z, Lin X, *et al.*. Camrelizumab plus rivoceranib versus sorafenib as first-line therapy for unresectable hepatocellular carcinoma (CARES-310): a randomised, open-label, international phase 3 study. The Lancet 2023;402(10408):1133-1146.
39. Shi YK, Wang L, Han BH, Li W, Yu P, Liu YP, *et al.*. First-line icotinib versus cisplatin/pemetrexed plus pemetrexed maintenance therapy for patients with advanced EGFR mutation-positive lung adenocarcinoma (CONVINCE): a phase 3, open-label, randomized study. Ann Oncol 2017;28(10):2443-2450.
40. Shi Y, Zhang L, Liu X, Zhou C, Zhang S, Wang D, *et al.*. Icotinib versus gefitinib in previously treated advanced non-small-cell lung cancer (ICOGEN): a randomised, double-blind phase 3 non-inferiority trial. The lancet oncology 2013;14(10):953-961.
41. Lu S, Dong X, Jian H, Chen J, Chen G, Sun Y, *et al.*. AENEAS: A Randomized Phase III Trial of Aumolertinib Versus Gefitinib as First-Line Therapy for Locally Advanced or

MetastaticNon-Small-Cell Lung Cancer With EGFR Exon 19 Deletion or L858R Mutations. J Clin Oncol 2022;40(27):3162-3171.
42. Zhou C, Chen G, Huang Y, Zhou J, Lin L, Feng J, *et al.*. Camrelizumab plus carboplatin and pemetrexed versus chemotherapy alone in chemotherapy-naive patients with advanced non-squamous non-small-cell lung cancer (CameL): a randomised, open-label, multicentre, phase 3 trial. Lancet Respir Med 2021;9(3):305-314.
43. Huang J, Xu J, Chen Y, Zhuang W, Zhang Y, Chen Z, *et al.*. Camrelizumab versus investigator's choice of chemotherapy as second-line therapy for advanced or metastatic oesophageal squamous cell carcinoma (ESCORT): a multicentre, randomised, open-label, phase 3 study. The Lancet Oncology 2020;21(6):832-842.
44. Yang Y, Qu S, Li J, Hu C, Xu M, Li W, *et al.*. Camrelizumab versus placebo in combination with gemcitabine and cisplatin as first-line treatment for recurrent or metastatic nasopharyngeal carcinoma (CAPTAIN-1st): a multicentre, randomised, double-blind, phase 3 trial. The Lancet Oncology 2021;22(8):1162-1174.
45. Luo H, Lu J, Bai Y, Mao T, Wang J, Fan Q, *et al.*. Effect of camrelizumab vs placebo added to chemotherapy on survival and progression-free survival in patients with advanced or metastatic esophageal squamous cell carcinoma: the ESCORT-1st randomized clinical trial. Jama 2021;326(10):916-925.
46. Ren S, Chen J, Xu X, Jiang T, Cheng Y, Chen G, *et al.*. Camrelizumab plus carboplatin and paclitaxel as first-line treatment for advanced squamous NSCLC (CameL-Sq): a phase 3 trial. Journal of Thoracic Oncology 2022;17(4):544-557.
47. Qin S, Chan SL, Gu S, Bai Y, Ren Z, Lin X, *et al.*. Camrelizumab plus rivoceranib versus sorafenib as first-line therapy for unresectable hepatocellular carcinoma (CARES-310): a randomised, open-label, international phase 3 study. The Lancet 2023;402(10408):1133-1146.
48. Verma S, Miles D, Gianni L, Krop IE, Welslau M, Baselga J, *et al.*. Trastuzumab emtansine for HER2-positive advanced breast cancer. New England journal of medicine 2012;367(19):1783-1791.
49. Mai H, Chen Q, Chen D, Hu C, Yang K, Wen J, *et al.*. Toripalimab or placebo plus chemotherapy as first-line treatment in advanced nasopharyngeal carcinoma: a multicenter randomized phase 3 trial. Nature medicine 2021;27(9):1536-1543.
50. Wang Z, Cui C, Yao J, Zhang Y, Li M, Feng J, *et al.*. Toripalimab plus chemotherapy in treatment-naïve, advanced esophageal squamous cell carcinoma (JUPITER-06): A multi-center phase 3 trial. Cancer cell 2022;40(3):277-288. e3.
51. Wang Z, Wu L, Li B, Cheng Y, Li X, Wang X, *et al.*. Toripalimab plus chemotherapy for patients with treatment-naive advanced non－small-cell lung cancer: a multicenter randomized phase III trial (CHOICE-01). Journal of Clinical Oncology 2023;41(3):651.
52. Xu B, Yan M, Ma F, Hu X, Feng J, Ouyang Q, *et al.*. Pyrotinib plus capecitabine versus lapatinib plus capecitabine for the treatment of HER2-positive metastatic breast cancer (PHOEBE): a multicentre, open-label, randomised, controlled, phase 3 trial. The Lancet Oncology 2021;22(3):351-360.
53. Qin S, Bi F, Gu S, Bai Y, Chen Z, Wang Z, *et al.*. Donafenib versus sorafenib in first-line treatment of unresectable or metastatic hepatocellular carcinoma: a randomized, open-label, parallel-controlled phase II-III trial. Journal of Clinical Oncology 2021;39(27):3002.
54. Lin Y, Qin S, Yang H, Shi F, Yang A, Han X, *et al.*. Multicenter randomized double-blind phase III trial of donafenib in progressive radioactive iodine-refractory differentiated thyroid cancer. Clinical

Cancer Research 2023;29(15):2791-2799.
55. Goetz MP, Toi M, Campone M, Sohn J, Paluch-Shimon S, Huober J*, et al.*. MONARCH 3: abemaciclib as initial therapy for advanced breast cancer. J clin oncol 2017;35(32):3638-3646.
56. Sledge Jr GW, Toi M, Neven P, Sohn J, Inoue K, Pivot X*, et al.*. MONARCH 2: abemaciclib in combination with fulvestrant in women with HR+/HER2− advanced breast cancer who had progressed while receiving endocrine therapy. Journal of clinical oncology 2017;35(25):2875-2884.
57. Zhang P, Zhang Q, Tong Z, Sun T, Li W, Ouyang Q*, et al.*. Dalpiciclib plus letrozole or anastrozole versus placebo plus letrozole or anastrozole as first-line treatment in patients with hormone receptor-positive, HER2-negative advanced breast cancer (DAWNA-2): a multicentre, randomised, double-blind, placebo-controlled, phase 3 trial. The Lancet Oncology 2023;24(6):646-657.
58. Xu B, Zhang Q, Zhang P, Hu X, Li W, Tong Z*, et al.*. Dalpiciclib or placebo plus fulvestrant in hormone receptor-positive and HER2-negative advanced breast cancer: a randomized, phase 3 trial. Nature medicine 2021;27(11):1904-1909.
